# A Phase II randomised controlled trial assessing the feasibility, acceptability and potential effectiveness of Dignity Therapy for older people in care homes: Study protocol

**DOI:** 10.1186/1471-2318-9-9

**Published:** 2009-03-24

**Authors:** Sue Hall, Harvey Chochinov, Richard Harding, Scott Murray, Alison Richardson, Irene J Higginson

**Affiliations:** 1 Department of Palliative Care, Policy & Rehabilitation, King's College London, Weston Education Centre, Cutcombe Road, London, SE5 9RJ, UK; 2 Manitoba Palliative Care Research Unit, CancerCare Manitoba, Department of Psychiatry, University of Manitoba, 3017-675 McDermot, Winnipeg, MB R3E 0V9, Canada; 3 General Practice Section, Division of Community Health Sciences, The University of Edinburgh, Levinson House, 20 West Richmond Street, Edinburgh, EH8 9DX, UK; 4 The Florence Nightingale School of Nursing and Midwifery, King's College London, James Clerk Maxwell Building, 57 Waterloo Road, London, SE1 8WA, UK

## Abstract

**Background:**

Although most older people living in nursing homes die there, there is a dearth of robust evaluations of interventions to improve their end-of-life care. Residents usually have multiple health problems making them heavily reliant on staff for their care, which can erode their sense of dignity. Dignity Therapy has been developed to help promote dignity and reduce distress. It comprises a recorded interview, which is transcribed, edited then returned to the patient, who can bequeath it to people of their choosing. Piloting has suggested that Dignity Therapy is beneficial to people dying of cancer and their families. The aims of this study are to assess the feasibility, acceptability and potential effectiveness of Dignity Therapy to reduce psychological and spiritual distress in older people reaching the end of life in care homes, and to pilot the methods for a Phase III RCT.

**Methods/design:**

A randomised controlled open-label trial. Sixty-four residents of care homes for older people are randomly allocated to one of two groups: (i) Intervention (Dignity Therapy offered in addition to any standard care), and (ii) Control group (standard care). Recipients of the "generativity" documents are asked their views on taking part in the study and the therapy. Both quantitative and qualitative outcomes are assessed in face-to-face interviews at baseline and at approximately one and eight weeks after the intervention (equivalent in the control group). The primary outcome is residents' sense of dignity (potential effectiveness) assessed by the Patient Dignity Inventory. Secondary outcomes for residents include depression, hopefulness and quality of life. In view of the relatively small sample size, quantitative analysis is mainly descriptive. The qualitative analysis uses the Framework method.

**Discussion:**

Dignity Therapy is brief, can be done at the bedside and could help both patients and their families. This detailed exploratory research shows if it is feasible to offer Dignity Therapy to residents of care homes, whether it is acceptable to them, their families and care home staff, if it is likely to be effective, and determine whether a Phase III RCT is desirable.

**Trial registration:**

Current Controlled Clinical Trials: ISRCTN37589515

## Background

Although most older people living in nursing homes die there, there is a dearth of good quality evaluations of interventions to improve their end of life care [1]. Residents usually have multiple health problems, making them heavily reliant on staff for their care, which can erode their sense of dignity. Maintaining dignity is given a high priority in health and social care strategy documents in most European countries, and particular concerns have been raised about loss of dignity in care [2]. Loss of dignity for people reaching the end of their lives is associated with high levels of psychological and spiritual distress and the loss of the will to live [3]. Pride, self-respect, quality of life, well-being, hope and self-esteem have been shown to be associated with dignity. For some people, a sense that nothing of one's life will be transcendent of death was associated with loss of dignity, and many felt that maintaining dignity was highly dependent on how they perceived themselves to be seen by others. Chochinov's study also found that in-patients were more likely to suffer from loss of dignity.

An empirically-based model of dignity has been developed from interviews with hospice patients [4], which forms the basis of a brief psychotherapy to help promote dignity and reduce distress at the end of life [5]. The therapy addresses physical, psychosocial, existential and spiritual domains of concern or distress. It is brief, can be done at the bedside and aims to help both patients and their families. It comprises an interview with a trained therapist (usually a nurse or other health care professional), which is recorded, transcribed, edited then returned to the patient, who, when they are satisfied with it, can share or bequeath the resulting "generativity" document to people of their choosing. A preliminary evaluation of Dignity Therapy conducted with hospice patients in Canada produced positive findings for patients [5] and their families [6]. Randomised controlled trials of Dignity Therapy for hospice patients are underway in Canada, Australia and the USA, however, their findings may not be generalisable to those with a diagnosis other than cancer or the older people reaching the end of life in care homes.

An exploratory study of the views of older people in care homes on maintaining dignity supported many of the constructs in Chochinovs's dignity model, suggesting that Dignity Therapy has the potential to be of benefit to older people in care homes [7]. However, this is a very different context to that in which Dignity Therapy was developed, namely, specialist palliative care. This is likely to impact on the feasibility, acceptability and effectiveness of the intervention. One important difference to be considered is the high prevalence of cognitive problems in residents, the majority of whom are following a frailty rather than cancer illness trajectory. In-depth piloting which includes an exploration of the feasibility of delivering an intervention and attention to the context in which interventions take place, as planned here, is recommended in the new Medical Research guidance for developing and evaluating complex interventions [8]. As recommended in the MRC framework, we will also test our proposed outcome measures. The results will inform the design of a Phase III randomised controlled trial.

## Aims and objectives

The aims of the study are to assess the feasibility, acceptability and potential effectiveness of Dignity Therapy to reduce psychological and spiritual distress in older people in care homes. The specific objectives are to:

a) Determine whether Dignity Therapy is likely to increase peoples' sense of dignity or reduce psychological or spiritual distress.

b) Determine whether it is feasible to provide Dignity Therapy to older people in care homes.

c) Determine whether Dignity Therapy is acceptable to residents and their families.

d) Pilot methods for a larger (Phase III) randomized controlled trial (e.g. recruitment, randomization, follow-up, suitability of measures).

## Methods

### Study design

A Phase II open-label randomized controlled trial comprising two groups: (i) Intervention (Dignity Therapy offered in addition to standard psychological care), and (ii) Control group (standard psychological care). Consenting participants have been randomly allocated to one of these two groups after baseline (T1) measures have been collected. Standard psychological care will vary between care homes. None are providing Dignity Therapy. The nature of such care is being collected as part of this study.

### Randomization

Randomization was conducted by an independent statistician. Treatment allocation (Dignity Therapy or control) was performed by block randomization with a fixed block size of two. Allocation concealment is facilitated by using sequentially numbered opaque sealed envelopes for consecutive and eligible participants. To reduce the risk of bias, the research assistant opens the next envelope to ascertain which group the resident has been allocated to after baseline measures have been collected from participants.

### Ethical Approval

This study has been approved by The Joint South London and Maudsley and the Institute of Psychiatry NHS Research Ethics Committee (24/11/2008, ref: 08/H0807/75), and meets the requirements of the local Research Governance Framework.

### Participants

The sample will comprise 64 residents in care homes in South East London (Lambeth, Southwark & Lewisham). Based on our recent experiences of recruiting residents of care homes, we expect to recruit approximately 10 residents in each care home. Consequently we have opportunistically recruited six care homes to the study. These are local homes in which we have conducted research in the past which expressed an interest in Dignity Therapy. We will approach more care homes if this becomes necessary. Characteristics of care homes (size, ownership, registration, standard care) will be recorded and reported.

#### Inclusion criteria

Residents aged 65 years old or over are included. Not all residents have a "terminal" illness and they are not selected on the basis of receiving palliative care. However, all residents in nursing homes are frail and could be considered as reaching the end of life phase. Participants are not screened for spiritual or psychological distress, or loss of dignity, however, these are assessed at baseline to explore the potential moderating effects of these variables on the impact of the intervention.

#### Exclusion criteria

Residents are excluded if care home managers feel they are too ill to be interviewed, or unable to provide informed consent either due to cognitive problems, or to the severity of their illness, or because they are unable to understand English. Residents with moderate or severe cognitive impairment are excluded since their "generativity" documents are likely to reflect a fractured sense of self, which could be distressing to them and recipients of these documents.

Recipients of the Dignity Therapy documents (usually family or friends) are invited into the study to give their views on taking part in the study and the therapy.

### Sample size calculations

We are obtaining in-depth information on taking part in the study and receiving therapy from a relatively small sample, rather than aiming to detect significant group differences. One of the aims of the study is to estimate the effect size for an RCT. Allowing for a 25% loss at follow-up, a sample of 64 will give us 77% power to detect an effect size of 0.80 standard deviation units in the Dignity Inventory [9] between the intervention and control groups at the (two-tailed test) 5% level of significance. This will be sufficient to estimate recruitment, follow-up rates and the sample size for a Phase III trial, and to explore qualitative outcomes.

### Intervention group

The therapy is delivered by a palliative care nurse who has been trained in Dignity Therapy by Harvey Chochinov (who developed it). Training included the theoretical basis for the intervention, demonstrations of Dignity Therapy, a detailed overview of the manual, editing the Dignity Therapy documents and working with residents to produce a document that will be helpful for them and its recipients.

A standard framework of questions used in the therapy is given to residents in the intervention group (after randomisation) to give them the opportunity to think about their responses before the session (Table 1). The question framework provides a flexible guide for the nurse delivering the therapy (the therapist) to shape the interview, based on patients' level of interest and responses. The therapist follows the residents' cues, helping them to structure and organise their thoughts, for example, by asking questions about time sequences, how events are causally related to each other and facilitating the disclosure of thoughts, feelings and memories. These interviews are tape-recorded, quickly transcribed verbatim then shaped into a narrative using a formatted editing process. This includes clarifications (eliminating colloquialisms, non-starters and sections not related to the "generativity" material, such an interruptions), chronological corrections, tagging and editing any content that might inflict significant harm on recipients of the document (after discussion with the resident) and finding a suitable ending for the document which is appropriate to the residents' overall message. Another session is arranged for the therapist to read the edited transcript to the participants, who are invited to make any editorial suggestions, including identifying errors of omission or commission. Once these "generativity" documents are finalised, they are given to residents to share with anyone they choose. There is no evidence from previous studies that this has been problematic.

**Table 1:** Framework of questions used in Dignity Therapy

• Tell me a little about your life history; particularly the parts that you either remember most, or think are the most important? Another way of putting this, which may elicit answers from some residents, is to ask, when did you feel most alive?
• Are their particular things that you would want your family to know about you, and are their particular things you would want them to remember?

• What are the most important roles you have played in your life (family roles, vocational roles, community service roles, etc)? Why were they so important to you, and what do you think you accomplished within those roles?

• What are your most important accomplishments, and what do you feel most proud of?

• Are there particular things that you feel still need to be said to your loved ones, or things that you would want to take the time to say once again?

• What are your hopes and dreams for your loved ones?

• What have you learned about life that you would want to pass along to others? What advise or words of guidance would you wish to pass along to your [son, daughter, husband, wife, parents, other(s)]?

• Are their words or perhaps even instructions you would like to offer your family, in order to provide them with comfort or solace?

• In creating this permanent record, are their other things that you would like included?

Since residents' conditions can fluctuate rapidly, the timing of the contacts can be relaxed and meetings rescheduled. If a resident's condition deteriorates, meetings are rescheduled up to three times before sensitively withdrawing them from the study. The therapist makes detailed notes of her experiences of giving each intervention and any deviations from the protocol. One in three therapy transcripts is randomly selected for review by the principal investigators. A quality assurance protocol has been developed to assess adherence to the Dignity Therapy protocol and deviations from the protocol will be reported as part of the feasibility study.

### Control group

Residents in the control group have at least three interviews with the research assistant. Completing the measures and taking part in the interview gives them an opportunity to talk about their feelings. The extent to which they feel that this is therapeutic is explored in the interviews.

### Recruitment Procedure

**Figure 1:**
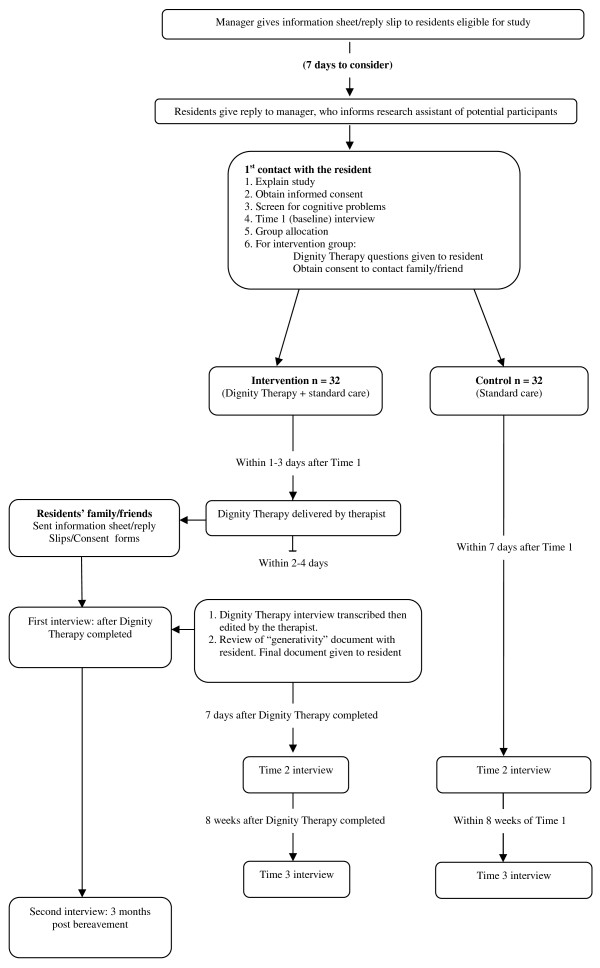
**Recruitment and follow-up procedure**.

The recruitment and follow-up procedure is shown in Figure 1. Care home managers are asked to distribute patient information sheets and reply slips (indicating whether or not they are interested in taking part in the study) to all residents eligible for the study. At least one week is given for full consideration and discussion of participation in the study with family and friends. Residents are asked to give their reply slips to the care home manager who gives them to the research assistant. The research assistant organises a convenient time to meet with the resident. At this meeting the research assistant: (i) explains the study to the resident, (ii) answers any questions they may have about their participation in the study, (iii) checks that they have fully understood the remit and implications of the study before obtaining written consent and proceeding with the Time 1 (baseline) interview. As a final check residents are screened with the Blessed Orientation Memory Concentration Test [10] to assess cognitive functioning. It has been suggested by Chochinov (personal communication) that Dignity Therapy is not suitable for residents with a score equal to or greater than 15. In such cases residents are excluded. This is done sensitively: the research assistant spends some time chatting with them about neutral topics. This approach has worked successfully in a previous study involving residents of care homes [7]. The proportion of residents excluded at this stage will be reported.

The research assistant collects baseline data from all residents taking part in the study (T1), then opens the next envelope in sequence to ascertain group allocation. Residents in the control group are reminded that they will still have the opportunity to talk to the researcher about how they are feeling and about their views on taking part in the study. Residents in the intervention groups are given the framework of therapy questions (Table 1) so that they have an opportunity to think about their answers before the therapy interview. The research assistant arranges a time for the therapist to conduct the therapy interview (usually within a couple of days). She also leaves a picture of the therapist with the participant information sheet and the date and time of the therapy session (session reminder card) so that the resident is not confused when visited by another person. The therapist arranges a mutually convenient time for the therapy visits. Although these documents can be bequeathed once the participant has died, they can also be given to people of their choosing at any time. If they wish, residents can choose to keep the document for themselves and not bequeath it. Care home managers keep a list of all the residents who have "generativity" documents to ensure they are sent to recipients once the resident has died. The nurse leaves a session reminder card with the resident as a reminder of when the research assistant will call back to arrange the T2 interview (approximately one week later).

At recruitment the research assistant asks residents in the intervention group to nominate a family member or friend who visits them regularly in the care home. With the resident's permission, the nominated family member or friend is invited to take part in a telephone interview once Dignity Therapy has been completed. The nominated family member or friend is often the recipient of the "generativity" document. Residents are not excluded if they do not nominate a family member. Should they wish to do so, they can chose to leave their "generativity" document with their personal effects rather than to a named person. If the resident mentions that they wish to bequeath to someone other than the nominated family member or friend we will seek permission from the resident to also invite them for interview. The research assistant follows up with the care home to determine when the resident has died. We complete interviews three months post bereavement with all nominated family and friends and any additional recipients of the document.

### Outcome measures

Both quantitative and qualitative outcomes are assessed. These are collected from residents by face-to-face interviews at two time-points: approximately one (T2) and eight weeks (T3) after the dignity document has been completed, and the equivalent in the control group. Longer-term follow-up has not been included in the previous evaluation of Dignity Therapy, or in the current trials being conducted by Chochinov and colleagues. However, we believe it is important to evaluate the longer-term impact of the intervention. The feasibility of doing this is explored as part of this feasibility study. Quantitative measures have been validated and used with older people and are fairly quick and easy to use. Qualitative interview schedules have been developed for the study.

#### Main outcome for residents

The primary outcome is residents' sense of dignity (potential effectiveness). This will be assessed at baseline (T1) and at one week (T2) and eight weeks (T3) follow-up, using the Patient Dignity Inventory [9]. This measure evolved directly from the dignity model, therefore, questions correspond to each of the model themes and sub-themes, including: physical, psychosocial, existential and spiritual domains of concern or distress. This measure has been validated in Canada and has been shown to have excellent face, internal, test-retest and concurrent validity.

#### Secondary outcomes for residents

*Potential effectiveness *is also assessed using: the Geriatric Depression Scale [11] (psychological distress); the Herth Hope Index [12] (hopefulness); the EQ-5D [13], and a two item measure used by Harvey Chochinov in the current trials of Dignity Therapy (quality of life). With the consent of participants, we are conducting a detailed qualitative analysis of the therapy transcripts to provide insight into concerns which might impact on the effectiveness of the intervention.

To assess *feasibility *of delivering Dignity Therapy in this setting, time taken to organize and conduct the Dignity Therapy sessions, transcribe and edit narratives, deviations from the therapy protocol and uncompleted interventions and the reasons, and the therapist's perceptions of competence as a result of training are recorded.

To assess the *acceptability *of Dignity Therapy we are conducting semi-structured interviews with residents in the intervention group to obtain their views on the intervention. The therapist is recording her experiences of delivering the therapy and her observations of resident's responses during and after the therapy. As suggested by Ferrell [14], we are reporting case studies of any difficult cases.

#### Demographic measures

Demographic information is collected, including: cognitive functioning (using the Blessed Orientation Memory Concentration test, [10]), co-morbidity (using the Comorbidity Index and Scores of Charlson, [15]), performance status (using Karnosfsky scores, [16]) and ability to perform activities of daily living (Barthel scores, [17]), age, gender, ethnic group.

#### Outcome measures for family/friends/recipients of "generativity" documents

In completion of the therapy, semi-structured telephone interviews are conducted with residents' family/friends and other recipients of the "generativity" documents to obtain their perceptions of the impact of the intervention, on themselves and on the resident, and their views on taking part in the study. For residents who die in the data collection period of the study, follow-up telephone interviews are conducted with family/friends/recipients of the "generativity" documents at three months post bereavement. As in the initial interview, these cover their perceptions of the impact of the intervention, on themselves and on the resident, and their views on taking part in the study. They are also asked to complete the Complicated Grief Assessment [18], which is used to screen for complicated grief reactions. The questionnaire is mailed to them after the telephone interview along with a pre-paid envelope in which to return it. Their age, gender, ethnic group and relationship to residents are also recorded.

#### Piloting methods for a Phase III trial

Time taken to obtain informed consent and collect outcomes, exclusions, recruitment and drop-out rates (residents and their family and friends) are recorded. In addition their views on taking part in the study (e.g. being randomised) are sought in qualitative interviews, and their views on completing the outcome measures are recorded when they are completing them. Any problems with completing measures will be reported, including missing data.

### Analyses

#### Quantitative data

In view of the relatively small sample size, analyses will be mainly descriptive. However, between and within participant comparisons of outcomes will be conducted and the appropriate effect size estimates reported. The intervention and control group will be compared on the main outcome (a sense of dignity) and secondary outcomes (depression, hopefulness and quality of life). We will also compare Time 2 and Time 3 follow-up with baseline for both groups on these measures. The two groups will also be compared on demographic characteristics and baseline measures. If necessary these will be controlled for in the analyses. We will also report recruitment rates and compare drop-out rates and missing data in the two groups.

#### Qualitative data

The Framework method of analysis will be used [19]. Analyses will be both deductive (from pre-set aims and objectives) and inductive (arising from participants views). This method tends to be more structured than some other methods of qualitative analysis and the process more explicit and more informed by a priori questions. It is designed so that it can be more easily understood and assessed by people other than the analyst, such as funding bodies, policy makers and participants. Throughout the analytical process we will use strategies to maximise credibility, criticality, and authenticity[20]. The QSR NVIVO software package is being used to manage the data.

## Discussion

There is a dearth of rigorously evaluated interventions to reduce psychological and spiritual distress for older people in care homes. Dignity Therapy, a brief intervention, which can be done at the bedside and aims to help both patients and their families and could help bolster a sense of dignity and reduce distress for residents reaching the end of life in care homes. This detailed exploratory study will show if this intervention is likely to be effective, if it is acceptable to residents, their families and care home staff, and if it is feasible to offer it in this context.

As recommended by new Medical Research Council guidance, [8], we plan to explore how Dignity Therapy works in this new context, who it works for, and if it fails, why it fails and any unexpected consequences. Older people in care homes differ from those with advanced cancer with whom the intervention was developed. For example, they may be less concerned by thoughts of death and more concerned about the multiple losses they have experienced [7]. A substantial number will have varying degrees of cognitive impairments and sensory losses, and some may have no one to leave their "generativity" document to. We need to see if it is feasible to deliver Dignity Therapy to people with hearing impairments and explore its impact on people with minor cognitive impairments and/or those with no close friends or family. It is possible that residents with minor cognitive impairments may find the intervention taxing if they need to struggle to recall names of the people and places which have been important in their lives. It may be necessary to tailor Dignity Therapy to be used in this new context. Some residents, may decide they wish to give their "generativity" documents to care home staff, who often become substitute family. The impact on such recipients has not been investigated.

Conducting research with residents in care homes can also be challenging. For example, taking informed consent can be difficult with some residents. In previous studies we have had completed expression of interest forms from residents we have found were unable to understand their participation in the study. Excluding residents who have expressed an interest in taking part needs to be handled sensitively and takes time. Finding a private place to conduct interviews at a time when residents are not tired or engaged in other activities can be difficult, resulting in repeated visits and sometimes long periods of waiting. All these factors and more need to be explored for a Phase III RCT to be developed. However, if such a trial shows Dignity Therapy is effective for older people in care homes, it could be a relatively low cost intervention, which could be offered routinely by care homes.

## Competing interests

The authors declare that they have no competing interests.

## Authors' contributions

SH led the drafting of this paper. HC developed Dignity Therapy. SH & IJH co-conceived the study. All authors co-applied for funding and contributed to the development of the protocol and the final draft of this paper.

## Pre-publication history

The pre-publication history for this paper can be accessed here:


